# Basic research in orthopedics: India

**DOI:** 10.4103/0019-5413.55971

**Published:** 2009

**Authors:** Satish Chandra Goel

**Affiliations:** *Department of Orthopaedics, Institute of Medical Sciences, Banaras Hindu University, Varanasi, India*

Research is the backbone of science. The scientific revolution in every sphere of daily life, including orthopedics, during the last two centuries has been made possible due to extensive research in the field. We may be able to find an answer to the problems of genetic disorders, degenerative diseases, etc., due to the ongoing research.

Many surgical treatments have become outdated and are not based on firm scientific background. Majority of the methods for patient evaluation and treatment lack the scientific accuracy of prospective, double-blind studies. These problems can only be solved by a committed scientific research in the field of orthopedics. Unfortunately, very few orthopedic specialists, usually in academic hospitals, are actively involved in the scientific research.

Problems of economically poor countries and Asian countries are very different from those of the western world, hence the solutions offered have to be innovative. Could we allow squatting and sitting cross-legged in a lotus posture follow total knee arthroplasty?

Most of the conferences, workshops, and symposia are also based on implants and instruments and methods to use them. Very little is said about biology and basic sciences. This leads to the fear that our young orthopedic surgeons may become a technician rather than an orthopedic specialist. May be it is due to the lack of industry support, but there is a perceptible lack of interest in basics as well. Looking at the Indian Journal of Orthopaedics, it is rare to find an original research article being submitted from India and even that is confined to few centers.

Till now, very little basic research has been done in India due to various reasons, mainly due to the lack of infrastructure and finance. Since the time of independence, the health administrators had been putting all emphasis on the treatment and prevention of the disease, which was essential looking at the vast population and prevalence of infective diseases at that time. Now that has been controlled and modern treatment facilities have been created all over. There is a need of having a research cell in all medical institutions with facilities for basic research and animal experimentation. This will require an animal house with operating facilities, bone research laboratory with facilities for tissue culture, biomedical engineering laboratory, etc. Further collaboration with mechanical, chemical, ceramic, and metallurgical engineering will be desirable.

India has second largest population in the world and by present estimates it will be the largest in the next 30 years. Hence, the healthcare system in India will change drastically, and we must anticipate a tremendous transformation due to advances in science and technology. Government and other funding agencies should establish an Orthopaedic Research and Education Foundation which can distribute the funds to finance the salaries of full-time career researchers in institutions capable of providing the atmosphere that good research requires.

The Indian orthopedic community today has many problems to solve. We have to seriously consider whether we can expand the horizons of orthopedic science, whether we can make an impact upon world orthopedic research communities in coming decades, and whether we will continue to be accepted as a less developed nation. The Indian Orthopaedic Association started a Basic Science section in 2006. Three research fellowships were also started and in 2008, an Orthopedic Research Foundation was started to fund basic research. Efforts are on to form an Orthopaedic Research Society. But this is not enough. With more than 10,000 orthopedic surgeons in the country, there should be many centers with infrastructure available for this purpose.

There is a controversy as to who should do basic research, a clinician or a basic scientist with a PhD. A clinician may not find time for it; on the other hand, a basic scientist may be away from realities. In this era of evidence-based medicine, research has to be guided by a clinician, so that it forms the basis of clinical application.

There must be realistic integration of research and clinical careers. Not only must there be protected time for both basic and clinical research, but an environment must also exist in which these activities are valued by the peers.^1^,^2^

We have to see that we maintain good clinical practice and ethical standard. American Academy of Orthopaedic Surgeons^3^ has set the following standards for the research which must be followed:

- All research and academic activities must be conducted under conditions of full compliance with ethical, institutional, and government guidelines. Patients participating in research programs must have given full informed consent and retain the right to withdraw from the research protocol at any time.
- Orthopedic surgeons should not claim as their own intellectual property which is not theirs. Plagiarism or the use of others' work without attribution is unethical.
- The principal investigator of a scientific research project or clinical research project is responsible for all aspects of the research, including reporting. The principal investigator may delegate portions of the work to other individuals, but this does not relieve the principal investigator of the responsibility for the work conducted by other individuals.
- The principal investigator or senior author of a scientific report is responsible for ensuring that appropriate credit is given for contributions to the research described.

It is essential that research in orthopaedics continues and that we try our best to facilitate high quality research. New ideas from basic research will lead to breakthroughs in field of osteoarthritis and cartilage and other musculoskeletal tissues. Tissue engineering may help in alleviating osteoarthritis and costly treatments like joint replacements might be avoided. Newly available facilities like electron microscopy, DNA sequencing and the Genome, and tissue culture will help us unravel many mysteries and help us in clinical application.

We have to strive to form a structure for orthopaedic research in India which can accommodate international collaboration and student exchanges, the scientific education of orthopaedic residents and provide a forum for peer-review of research proposals and publications. This structure must be characterised by strong interactions between clinical and basic research. Orthopaedic science must be promoted as a basis for professional careers.
